# Prediction of All-Cause Mortality and Cardiovascular Outcomes Using Ambulatory Arterial Stiffness and Ankle-Brachial Indices in Patients with Acute Myocardial Infarction: A Prospective Cohort Study

**DOI:** 10.3390/jcm14134627

**Published:** 2025-06-30

**Authors:** Areti Koumelli, Konstantinos Konstantinou, Athanasios Sakalidis, Konstantinos Pappelis, Emmanouil Mantzouranis, Christina Chrysohoou, Petros I. Nihoyannopoulos, Dimitrios Tousoulis, Konstantinos Tsioufis

**Affiliations:** 1 First Department of Cardiology, School of Medicine, National and Kapodistrian University of Athens, Hippokration General Hospital, 11527 Athens, Greece; kostiskon@gmail.com (K.K.); asakalidis@gmail.com (A.S.);; 2 Department of Cardiology, Royal Brompton and Harefield Hospitals, Guy’s & St Thomas’ NHS Foundation Trust, London UB9 6JH, UK; 3 Second Department of Ophthalmology, School of Medicine, National and Kapodistrian University of Athens, Attikon Hospital, 12462 Athens, Greece

**Keywords:** acute myocardial infarction, ankle-brachial index, ambulatory arterial stiffness index, cardiovascular mortality, peripheral artery disease, risk stratification, hypertension, atherosclerosis, major adverse cardiovascular events, prognostic markers

## Abstract

**Background/Objectives**: The ankle-brachial index (ABI) is a non-invasive diagnostic tool for peripheral artery disease (PAD) and a marker of systemic atherosclerosis, predictive of cardiovascular (CV) events. The ambulatory arterial stiffness index (AASI), derived from 24-h blood pressure monitoring, also predicts CV morbidity and mortality, particularly stroke. However, their combined prognostic utility in acute myocardial infarction (AMI) remains underexplored. This study aimed to assess the predictive value of ABI and AASI in patients with AMI. **Methods**: We conducted a single-center observational cohort study including 441 consecutive patients with AMI (79% male; mean age 62 years). ABI was measured using an automated device, with ≤0.9 defined as abnormal. AASI was calculated from 24-h blood pressure recordings. The primary endpoint was a composite of all-cause and CV death and major CV events, assessed in-hospital and over a 3-year follow-up. **Results**: Median ABI was 1.10 (IQR 1.00–1.18); 10.4% had abnormal ABI. Abnormal ABI was associated with a threefold higher risk of in-hospital adverse events (OR 2.93, 95% CI: 1.48–5.81, *p* = 0.002). In Cox regression, abnormal ABI predicted long-term all-cause mortality (HR 2.88, 95% CI: 1.53–5.42, *p* = 0.001), independent of traditional risk factors. Each 0.1 increase in AASI was linked to a 21% higher risk of the composite outcome (*p* = 0.001) and 25% increased risk of recurrent AMI or urgent revascularization (*p* = 0.001). **Conclusions**: In this prospective cohort of patients with AMI, ABI and AASI were associated with adverse outcomes, suggesting their potential role in risk stratification. These exploratory findings require validation in larger, multicenter cohorts to assess their incremental prognostic value and generalizability.

## 1. Introduction

Atherosclerosis is a generalized chronic inflammatory disease that frequently affects different vascular territories. It is mainly characterized by thickening of the intimal and medial layers and loss of elasticity. Multisite artery disease (MSAD) is characterized by the simultaneous presence of clinically significant atherosclerotic lesions in at least two major vascular regions. MSAD ranges from 10 to 15% in patients with coronary artery disease (CAD) to 60 to 70% in patients with severe carotid stenosis or lower extremity arterial disease (LEAD) [1].

The ankle-brachial index (ABI) is a quick and easy method to confirm the diagnosis and assess the severity of peripheral artery disease in the lower extremities. It is associated with the presence of cardiovascular risk factors and it is an indicator of generalized atherosclerosis. Ankle-brachial index represents the ratio between the systolic blood pressure measured at the ankle and the brachial arteries [2]. Both the American College of Cardiology (ACC)/American Heart Association (AHA) and European Society of Cardiology (ESC) guidelines emphasize that a low ABI (≤0.90) serves as a marker of systemic atherosclerosis and is independently associated with heightened cardiovascular morbidity and mortality. An ankle-brachial index (ABI) of 0.90 or lower has been linked to over a twofold increase in the 10-year incidence of coronary events and both cardiovascular (CV) and all-cause mortality [3,4].

Another non-invasive index of arterial stiffness is the ambulatory arterial stiffness index (AASI), an index calculated from ambulatory 24-h blood pressure (BP) measurements. It is calculated as 1 minus the regression slope of diastolic BP (DBP) on systolic BP (SBP) during ambulatory measurements [5,6]. Evidence indicates that AASI is significantly related with markers of target organ damage. Among hypertensive patients, each standard deviation increase in AASI corresponds to twofold increase in the likelihood of microalbuminuria, carotid structural changes and hypertrophic remodeling of the left ventricle [7]. AASI also independently predicts cardiovascular morbidity and mortality, especially stroke, beyond traditional risk factors [8]. However, data on the prognostic role of ABI and AASI in patients hospitalized with acute myocardial infarction (AMI) remain limited. This study prospectively evaluated the prevalence and clinical significance of these indices in a large cohort of consecutive patients with AMI [9].

## 2. Materials and Methods

### 2.1. Study Design and Population

This was a single-center, prospective, observational cohort study with a 3-year follow-up, enrolling hospitalized patients diagnosed with acute myocardial infarction (AMI), either with or without ST-segment elevation (STEMI: ST-elevation myocardial infarction; NSTEMI: non–ST-elevation myocardial infarction). All consecutive patients were fully informed about the objectives of the study, and written informed consent was obtained.

Eligible patients were adults aged between 18 and 85 years. Exclusion criteria included cardiogenic shock, cerebrovascular events, severe valvular heart disease, chronic heart failure, cardiomyopathy, chronic renal failure with an estimated glomerular filtration rate (GFR) < 30 mL/min/1.73 m^2^, active malignancy, severe systemic disease, and psychiatric illness preventing sufficient cooperation (Table 1).

**Figure A1:**
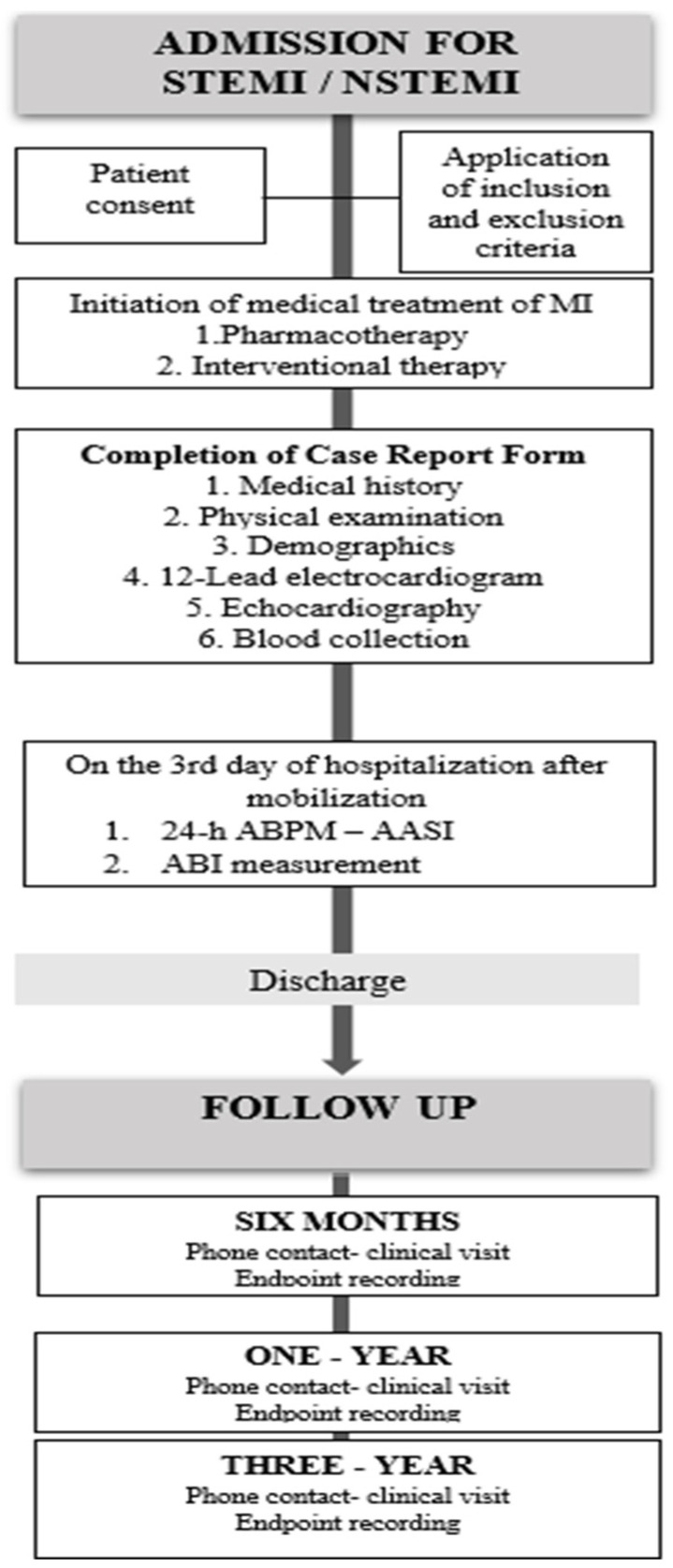
Study Design and Patient Follow-Up Protocol.

All participants received standard medical management for AMI and were followed during hospitalization, with subsequent scheduled follow-ups at 6, 12, and 36 months after the index event. (Figure A1).

### 2.2. Study Plan and Measurements

Given the observational nature of this study, patient management for AMI remained consistent with prevailing guidelines and institutional standards for both pharmacological and interventional treatment. On the day of admission, demographic data, cardiovascular risk factors, complete medical history of patients and baseline medications were meticulously collected on prespecified forms. During hospitalization, results from laboratory tests, the coronary angiography and echocardiography were collected. ABI and AASI measurements were obtained after the third day of hospitalization and at least 24 h following reperfusion and mobilization, provided the patient was clinically stable and receiving standard medical therapy, during a 24-h ambulatory blood pressure monitoring period. The ABI measured according to established methodology using a certified automated device ABPI MD, MESI^®^ [10,11,12]. An ABI value of 0.9 or less was considered abnormal. AASI was derived from the 24-h blood pressure recordings obtained with a Spacelabs Medical Model 90207 automatic non-invasive blood pressure monitor. Blood pressure data were analyzed using Spacelabs ABP Monitoring Software, version 2.00.03 (Spacelabs Medical).

### 2.3. Study Endpoints and Follow-Up

The prespecified follow-up period for each patient included monitoring during hospitalization (average hospital stay: 4 days) and subsequent follow-up for a total duration of 36 months. Throughout hospitalization and at 6, 12, and 36 months after the index event, patients and/or their relatives were contacted via telephone to complete a structured questionnaire documenting patient status and potential clinical endpoints. In cases of reported hospitalization, clinic visits were arranged to verify events through hospital records, while official death certificates were requested to confirm cause of death in deceased patients.

Over the 36-month follow-up, the primary endpoint consisted of a composite of fatal and non-fatal cardiovascular and renal events, including myocardial infarction (MI), unstable angina, coronary revascularization, admission due to decompensated heart failure, cerebrovascular events as stroke and transient ischemic attack (TIA), acute limb ischemia, peripheral vascular interventions, serum creatinine doubling and/or progression to end-stage renal disease. Secondary endpoints included individual evaluations of in-hospital clinical events, long-term cardiovascular and renal outcomes, cardiovascular death, and all-cause mortality (Table 2).

In-hospital major adverse cardiovascular events (MACE) were separately defined as a composite of all-cause mortality, MI (including type 4, type 5, and reinfarction), cardiogenic shock, acute pulmonary edema (PE), cerebrovascular events, and life-threatening arrhythmias. Atrial fibrillation (AF) occurring during hospitalization was recorded as a separate predefined event but was not included in the MACE composite.

Long-term outcomes assessed included all-cause and cardiovascular mortality, acute coronary syndrome (ACS)—defined as recurrent myocardial infarction (MI) or urgent coronary revascularization—stroke (ischemic, hemorrhagic, or transient), life-threatening arrhythmias, and rehospitalization for heart failure decompensation.

Event definitions followed established European Society of Cardiology (ESC) guidelines. Myocardial reinfarction, recurrent MI, cardiogenic shock, and pulmonary edema were classified according to ESC criteria [13]. Cerebrovascular events were defined as brain ischemia or hemorrhage confirmed by CT or MRI imaging. Life-threatening arrhythmias encompassed ventricular tachycardia, ventricular fibrillation, and complete heart block.

Of the 488 patients initially enrolled, 441 (90.4%) successfully completed the 36-month follow-up period, resulting in a dropout rate of 9.6%. Patients lost to follow-up were mainly due to withdrawal of consent, inability to establish contact despite repeated attempts, or relocation without updated information. These dropouts were evenly distributed across STEMI and NSTEMI groups, minimizing potential selection bias. All available patient data were included in the final analysis according to the intention-to-follow principle.

### 2.4. Statistical Analysis

#### Sample Size Calculation and Data Management

Continuous variables were described as mean ± standard deviation (SD) or median with interquartile range (IQR), depending on data distribution, while categorical variables were expressed as absolute numbers and percentages. Group comparisons were performed using parametric tests for normally distributed variables and non-parametric tests otherwise. Statistical significance was defined as a two-sided *p*-value ≤ 0.05, with adjustments for multiple comparisons made using the Bonferroni correction.

In-hospital events were assessed using multivariable binary logistic regression models to estimate odds ratios (ORs) with 95% confidence intervals (CIs). Longitudinal outcomes were analyzed using Cox proportional hazards models to derive hazard ratios (HRs) and 95% CIs.

In both cases, we originally built a full model, adjusted for the explanatory variables among age, sex, body mass index [BMI], SBP, DBP, smoking status, medication use, ACS type, previous MI, ejection fraction [EF], low-density lipoproteins [LDL], high-density lipoproteins [HDL], triglycerides [TG], troponin at presentation, peak troponin, and creatinine at presentation, that were statistically significant in univariable analysis. A stepwise backward elimination method was employed, sequentially removing the variable with the highest *p*-value until only significant predictors remained. We used ROC analysis and calculated Youden’s Index area under the curve (AUC) to determine optimal classification cutoffs for the in-hospital and longitudinal outcomes that were significantly associated with ABI.

Kaplan–Meier survival analyses were performed to evaluate time-to-event outcomes, including all-cause mortality and major cardiovascular events, over the 36-month follow-up period. Survival curves were generated for the overall cohort and predefined subgroups based on baseline ankle-brachial index (ABI) categories. Differences between survival curves were assessed using the log-rank test.

Sample size determination was based on expected event rates from the Global Registry of Acute Coronary Events (GRACE), targeting a 45% composite event rate at 6 months. A minimum sample size of 367 patients was initially estimated. To account for an anticipated 20–25% attrition rate due to missing data, patient withdrawal, or loss to follow-up, the final enrollment target was set at approximately 450 patients. This sample size was calculated to provide 80% power to detect significant intergroup differences with a two-sided alpha of 0.05 over the 36-month follow-up period.

All clinical endpoints and adverse events were systematically recorded in standardized case report forms and entered into a secure digital database, with rigorous electronic and on-site quality control procedures in place to ensure data integrity. Statistical analyses were performed using SPSS software, version 29 (IBM Corp., Armonk, NY, USA).

Sensitivity analyses were conducted to assess the robustness of the main findings. These included repeating multivariable models after excluding patients lost to follow-up, performing analyses stratified by ACS type (STEMI vs. NSTEMI), and adjusting for additional covariates such as hospitalization duration and revascularization status. Results remained consistent across all sensitivity analyses, reinforcing the stability of the associations observed.

## 3. Results

### 3.1. Characteristics of the Study Population

The baseline characteristics of the study cohort are presented in Table 3. Among the 441 patients enrolled, the mean age was 62 years, and 79.4% were male. Current smoking was reported in 52.4% of the population, and 67.6% had a history of arterial hypertension (AHT). An abnormal ABI ≤ 0.90 was identified in 10.4% of patients, and the median AASI was 0.43.

Coronary angiography revealed significant LM disease in 11.3% of patients and multivessel coronary disease in 51.7%. A significant lesion in the LM was more frequently observed among patients with NSTEMI compared to those with STEMI (15.6% vs. 7.9%, *p* = 0.011).

**Figure 1:**
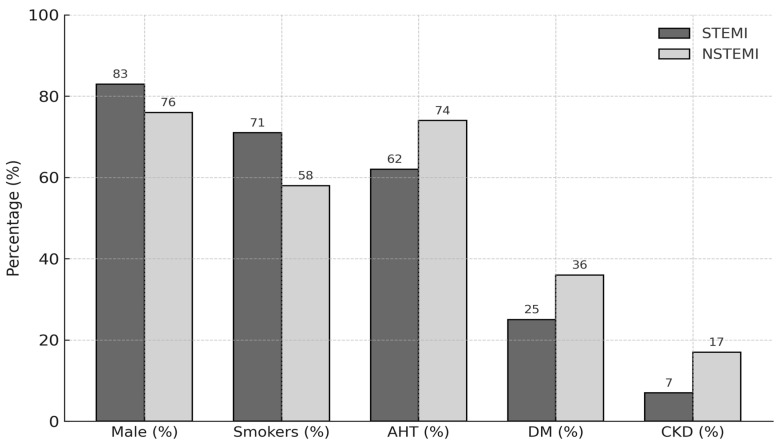
Clinical characteristics per type of acute myocardial infarction.

Patients presenting with NSTEMI were generally older and exhibited a higher prevalence of cardiovascular risk factors (Table 4, Figure 1). Notably, although the median ABI was similar between groups [STEMI: 1.10 (interquartile range [IQR]: 1.00–1.18) vs. NSTEMI: 1.10 (IQR: 1.00–1.17)], a significantly higher proportion of NSTEMI patients had an ABI < 0.90 (14.1% vs. 7.4%, *p* = 0.023), suggesting a greater burden of PAD in this subgroup.

Patients with abnormal ABI were significantly older [66 years (IQR: 57–78) vs. 61 years (IQR: 54–70), *p* = 0.04] and exhibited a markedly higher prevalence of CKD compared to those with normal ABI (26.1% vs. 9.6%, *p* < 0.001) (Table A1). No significant differences were observed between ABI groups regarding sex distribution, BMI, current smoking status, AHT, DM, dyslipidemia, or prior MI.

According to the subgroup analysis based on AMI type and ABI status, the study demonstrated the following findings:

Among patients with abnormal ABI no statistically significant differences were observed between STEMI and NSTEMI groups regarding most demographic and clinical characteristics, except for CKD, which was significantly more prevalent among NSTEMI patients (39.3% vs. 5.6%, *p* = 0.011) (Table A2).

A different pattern emerged among patients with an ABI > 0.9. In this subgroup, NSTEMI patients tended to be older [65 years (IQR: 57–74) vs. 60 years (IQR: 53–68), *p* < 0.001], had higher rates of arterial hypertension (73.7% vs. 61.6%, *p* = 0.012), diabetes mellitus (34.5% vs. 24.6%, *p* = 0.031), CKD (13.5% vs. 6.7%, *p* = 0.024), and were more frequently treated with antihypertensives, antidiabetics, and statins (Table A2). Moreover, they demonstrated impaired LV function with lower EF [50% (IQR: 40–55) vs. 45% (IQR: 37–50), *p* < 0.001] and higher peak creatinine levels during hospitalization [1.1 mg/dL (IQR: 0.9–1.4) vs. 1.0 mg/dL (IQR: 0.9–1.2), *p* = 0.018] (Table A3).

Coronary angiographic findings were generally comparable across STEMI and NSTEMI groups within both ABI categories. However, there was a non-significant trend toward greater LMA involvement in NSTEMI patients compared to STEMI patients, both in ABI ≤ 0.9 (25.0% vs. 5.6%, *p* = 0.089) and ABI > 0.9 groups (14.0% vs. 8.0%, *p* = 0.055) (Table A3).

### 3.2. In Hospital Outcomes

In the reduced multivariable logistic regression models, an abnormal ABI value was an independent predictor of the in-hospital composite outcome associated with a threefold increased risk [odds ratio (OR) 2.93, 95% confidence interval (CI): 1.48–5.81, *p* = 0.002] (Table A4).

Additionally, AASI demonstrated a significant association with atrial fibrillation onset, with every 0.1 increase translating into a 34% higher risk during hospitalization [OR 1.34, 95% CI: 1.12–1.60, *p* = 0.002] (Table A4).

### 3.3. Long Term Outcomes

In the multivariable Cox regression models, an abnormal ABI remained an independent predictor of all-cause mortality over the 36-month follow-up period [hazard ratio (HR) 2.88, 95% CI: 1.53–5.42, *p* < 0.001] (Table A5), independent of AHT, DM, LDL levels, and smoking status.

Regarding arterial stiffness, each 0.1 increase in AASI was associated with a 21% higher risk of the composite primary outcome [HR 1.21, 95% CI: 1.08–1.35, *p* = 0.001] and a 25% higher risk of developing a new ACS defined as recurrent MI and urgent coronary revascularization [HR 1.25, 95% CI: 1.09–1.43, *p* = 0.001] (Table A5).

**Figure 2:**
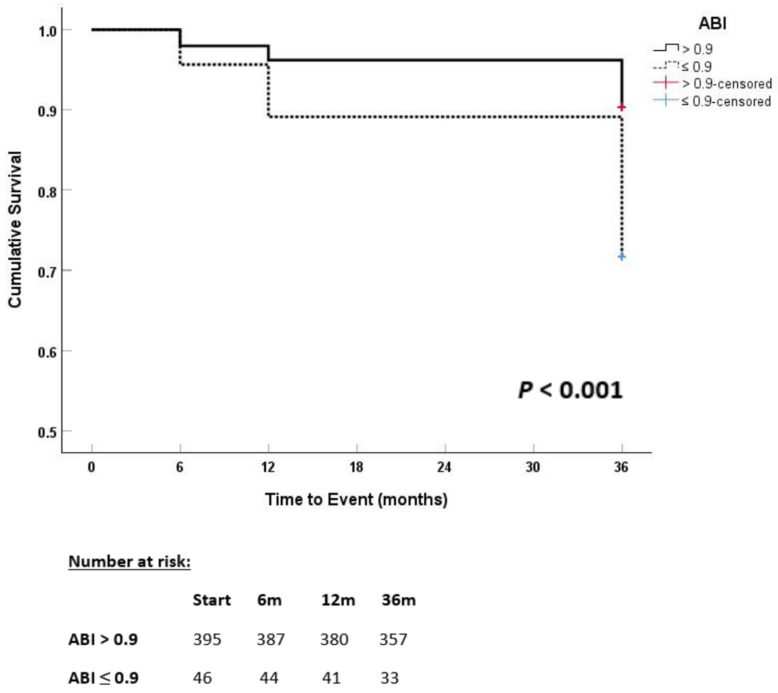
Kaplan–Meier Curves for All-Cause Mortality According to ABI Status.

In survival analysis, Kaplan–Meier curves demonstrated that patients with a pathological ABI had significantly lower cumulative survival rates compared to those with a normal ABI over the 36-month follow-up period (log-rank *p* < 0.001) (Figure 2). The divergence between the survival curves appeared early during follow-up and progressively widened over time, highlighting the strong prognostic impact of subclinical peripheral artery disease on long-term mortality.

**Figure 3:**
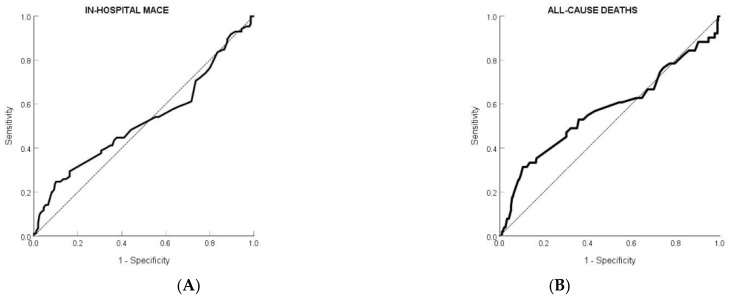
Receiver operator curves for predicting in-hospital major adverse cardiovascular outcomes (**A**) and all-cause deaths within 36 months (**B**) based on the ankle-brachial index (ABI).

We also performed ROC curve analysis to evaluate the discriminatory ability of ABI for predicting in-hospital MACE and long-term all-cause mortality. An ABI cutoff of <0.94 yielded the highest Youden Index for both outcomes. At this threshold, specificity was high—89.9% for in-hospital events and 89.5% for all-cause deaths—while sensitivity was more limited, at 24.7% and 31.4%, respectively. The corresponding AUC values were 0.525 and 0.571, indicating modest standalone discriminative performance (Table 5, Figure 3). Although the standalone discriminatory performance of ABI was limited, it remained statistically associated with adverse outcomes in multivariable models, underscoring its potential relevance when interpreted alongside other clinical variables.

**Figure 4:**
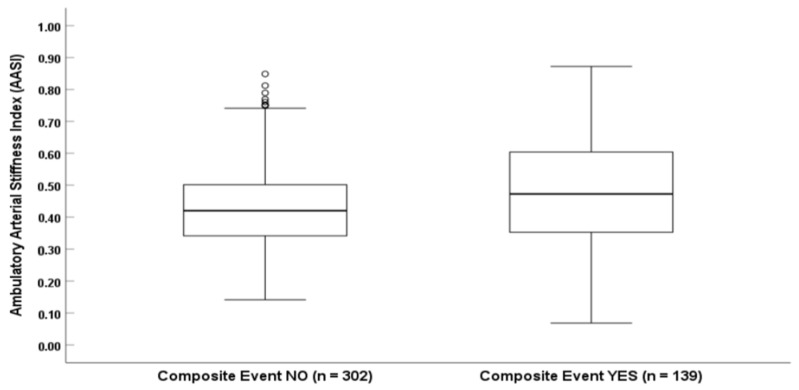
AASI According to Composite Outcome Status.

Furthermore, when analyzing arterial stiffness parameters, patients who experienced the composite primary outcome had significantly higher AASI values compared to those who remained event-free [median AASI 0.47 (IQR: 0.41–0.52) vs. 0.42 (IQR: 0.37–0.48), *p* < 0.001] (Figure 4). These findings underscore the prognostic value of ambulatory arterial stiffness in predicting adverse cardiovascular outcomes.

## 4. Discussion

This was the first study to investigate the relationship between two non-invasive tools, the ankle–brachial index and the ambulatory arterial stiffness index, with adverse cardiovascular and renal outcomes in patients hospitalized for acute myocardial infarction. Most previous studies examined ABI or AASI individually; in contrast, we evaluated both parameters simultaneously within the same population, providing a broader and more comprehensive assessment of vascular risk. This parallel evaluation, coupled with a prolonged follow-up, offers exploratory insights with potential clinical relevance.

Patients presenting with AMI often exhibit concurrent atherosclerotic conditions, such as peripheral artery disease. Growing evidence indicates that PAD is linked to worse long-term outcomes after AMI. Dinser et al. and Kirchberger et al. reported higher short- and long-term mortality rates in patients with AMI and PAD, while Attar et al. confirmed PAD as an independent predictor of adverse post-AMI outcomes [14,15,16]. These findings highlight the relevance of PAD in this clinical context.

The ABI Collaboration, which included 16 international cohort studies, demonstrated that a low ABI is a strong predictor of future cardiovascular disease. Likewise, AASI has been established through prior meta-analyses as a biomarker predictive of future cardiovascular events, stroke, and all-cause mortality in hypertensive populations [17,18].

Our study supports the prognostic significance of abnormal ABI values in patients hospitalized with AMI. An ABI ≤ 0.9 was strongly associated with long-term adverse cardiovascular outcomes, independent of traditional risk factors. These findings align with previous reports, including Ban et al., who demonstrated a significant association between low ABI and major adverse cardiovascular events in patients with AMI without a prior history of peripheral artery disease [19]. Similarly, Berkovitch et al. observed an incremental increase in MACE and one-year mortality in patients with low ABI and asymptomatic PAD [20].

Further supporting these findings, large cohort studies have shown consistent results. Xu et al. reported increased all-cause mortality below an ABI of 1.11, even within the normal range, while a Thai nationwide study found that ABI ≤ 0.9 was linked to a twofold higher mortality risk and greater renal decline [21,22]. Likewise, Masini et al. demonstrated increased mortality in patients with AMI and low ABI, independent of multivessel or carotid disease, and the PATHOS study linked abnormal ABI to a twofold higher one-year mortality in ACS patients [23,24]. A recent study by Verdoia et al. found that over 20% of patients with AMI undergoing percutaneous coronary intervention had an ABI ≤ 0.9 despite no prior diagnosis of PAD. Interestingly, higher platelet counts were independently associated with impaired ABI, suggesting a possible link between thrombotic burden and subclinical peripheral vascular disease. This adds an emerging pathophysiological dimension that warrants further exploration [25].

These data support the clinical utility of ABI not only in identifying PAD but as a broader prognostic tool in acute cardiovascular care.

In addition, we introduced the evaluation of AASI, highlighting arterial stiffness as a complementary risk marker. To our knowledge, this is one of the first studies to demonstrate a statistically significant association between AASI and adverse cardiovascular outcomes in AMI. While AASI has been identified as a prognostic marker in broader coronary artery disease populations, its role in AMI remains underexplored. In 2019, Cieślik-Guerra et al. evaluated 90 post-MI patients and reported that an AASI > 0.4235 was an independent predictor of cardiovascular death, recurrent infarction, unstable angina, coronary artery bypass surgery, and implantable cardioverter-defibrillator placement for heart failure [26]. Our findings are consistent with that study and extend its observations to a larger cohort.

We found no significant sex-based differences in long-term survival, consistent with previous epidemiologic data [27].

In light of these findings, incorporating additional non-invasive tools—such as multimodality imaging—could further enhance prognostic accuracy in patients with AMI. Recent studies have underscored the value of advanced imaging techniques, including cardiac MRI, echocardiography, and nuclear imaging, in providing comprehensive assessments of myocardial viability, infarct size, and left ventricular function.

Canton et al. highlighted the pivotal role of multimodality imaging in ischemic heart disease, emphasizing its ability to guide revascularization decisions by assessing myocardial viability [28]. Their findings suggest that integrating functional imaging into clinical pathways may improve patient outcomes through more individualized therapeutic strategies. Furthermore, they demonstrated that combining anatomical and functional imaging modalities can detect subtle myocardial dysfunction that may be overlooked with standard evaluations. This aligns with a growing body of literature advocating for multimodality imaging as a key component in advanced risk stratification.

Bergamaschi et al. further supported this approach by emphasizing the diagnostic and prognostic value of non-invasive anatomical and functional imaging in patients with chronic coronary syndromes [29]. Complementing these findings, a meta-analysis by Lipinski et al. confirmed the prognostic utility of stress cardiac MRI in individuals with suspected or known coronary artery disease, reinforcing its role alongside vascular markers in comprehensive risk assessment [30].

While these modalities are highly informative, they may not always be readily accessible. In contrast, ABI and AASI are cost-effective, non-invasive bedside tools that can be used routinely to support early post-AMI risk assessment.

Taken together, our findings provide early evidence that ABI and AASI serve as independent markers of long-term prognosis in AMI. Nonetheless, validation in larger, multicenter studies is essential before ABI and AASI can be routinely integrated into guideline-directed AMI risk stratification.

## 5. Limitations

This study presents several limitations, the foremost being its single-center design, which may reduce the generalizability of the results to broader populations. Therefore, larger multicenter studies are warranted to validate and extend our observations. Second, although the sample size was determined based on an a priori power analysis, it remains relatively modest, and larger cohorts would provide greater statistical power to detect more subtle associations. The heterogeneity in predictor-outcome associations across models may reflect underlying confounding or insufficient power and should be interpreted with caution. Third, the exclusion of patients with severe cardiac, renal, or systemic disease may have influenced outcome rates, particularly in-hospital event rates, potentially underestimating the burden of adverse outcomes in a real-world setting. Fourth, although our study focused on arterial stiffness using AASI, we did not evaluate arterial elastance, an important parameter of vascular load. Notably, a straightforward and noninvasive method for estimating effective arterial elastance—defined as the ratio of end-systolic pressure to stroke volume index—has been proposed and validated in a previous study, and including this parameter in future research may offer complementary insights into vascular-ventricular interaction [31]. Additionally, different classes of antihypertensive medications have been shown to exert variable effects on arterial stiffness, which may represent an unmeasured confounder in the relationship between AASI and clinical outcomes [32]. Finally, the study population was predominantly composed of white/Caucasian individuals, potentially limiting the external validity of our findings across different racial and ethnic groups.

## 6. Conclusions

In conclusion, our study suggests a potential prognostic value of non-invasive vascular markers in patients hospitalized with acute myocardial infarction. The ankle–brachial index may serve as a widely available, cost-effective, and non-invasive tool to assist in early risk identification. Furthermore, increased arterial stiffness, as reflected by the ambulatory arterial stiffness index, was associated with adverse outcomes, suggesting a potential complementary role in risk assessment. However, given the single-center design, limited sample size, and heterogeneity of predictor-outcome relationships, these findings should be interpreted with caution. The observed associations warrant further investigation before clinical applicability can be established. Overall, this study provides exploratory data that may inform the design of future multicenter investigations aimed at refining early post-infarction risk stratification.

## Figures and Tables

**Table 1:** Exclusion criteria.

1. Unstable angina
2. Acute myocardial infarction type II
3. Cardiogenic shock
4. Systolic blood pressure < 90 mmHg at baseline
5. Chronic renal failure with an estimated glomerular filtration rate < 30 mL/min/1.73 m^2^
6. Secondary hypertension
7. Severe valvular disease
8. Preexisting heart failure with reduced ejection fraction
9. Cardiomyopathy
10. Stroke
11. Transient Ischaemic Attack
12. Severe systemic disease
13. Active malignancy
14. History of peripheral artery disease
15. Psychiatric illness that prevents sufficient co-operation
16. Consent denial or withdrawal

**Table 2:** Secondary outcomes.

All-cause mortality
Cardiovascular death
Myocardial infarction (MI)
Unstable angina (UA)
Coronary revascularization
Stroke
Transient ischemic attack (TIA)
Hospitalization for heart failure
Life-threatening arrhythmias
Acute limb ischaemia
Peripheral revascularization procedure
Renal injury/failure

MI: Myocardial infraction, UA: Unstable angina, TIA: Transient ischaemic attack.

**Table 3:** Demographics of the study population (*n* = 441).

Age, years [median (IQR)]	62 (55 to 71)
Sex, % female	20.6
BMI [median (IQR)]	28.4 (25.4 to 32.0)
Smoking (current), %	52.4
Smoking (anytime), %	65.1
AHT, %	67.6
AHT medication use, %	61.9
DM, %	29.7
DM medication use, %	22.9
DLD, %	65.1
Statin use, %	39.9
ACS type, % STEMI	54.9
Previous MI, %	21.1
CVD family history, %	27.2
ASA use, %	28.3
Chronic heart disease, %	28.8
Heart failure, %	11.3
CKD, %	11.3

IQR, interquartile range; BMI, body mass index; AHT, arterial hypertension; DM, diabetes mellitus; DLD, dyslipidaemia; ACS, acute coronary syndrome; STEMI, ST-elevation myocardial infarction; MI, myocardial infarction; CVD, cardiovascular disease; ASA, acetylsalicylic acid; CKD, chronic kidney disease.

**Table 4:** Demographics per type of acute coronary syndrome.

	STEMI (*n* = 242)	NSTEMI (*n* = 199)	*p* Value
Age, years [median (IQR)]	60 (53 to 68)	66 (56 to 75)	<0.001
Sex, % female	17.4	24.6	0.061
BMI [median (IQR)]	28.4 (25.5 to 32.9)	28.3 (24.8 to 31.2)	0.13
Smoking (current), %	57.4	46.2	0.019
Smoking (anytime), %	70.7	58.3	0.007
AHT, %	62.0	74.4	0.006
AHT medication use, %	54.5	70.9	<0.001
DM, %	24.8	35.7	0.013
DM medication use, %	15.7	31.7	<0.001
DLD, %	62.4	68.3	0.19
Statin use, %	33.5	47.7	0.002
Previous MI, %	15.3	28.1	<0.001
CVD family history, %	31.0	22.6	0.049
ASA use, %	17.8	41.2	<0.001
Chronic heart disease, %	21.1	38.2	<0.001
Heart failure, %	7.0	16.6	0.002
CKD, %	6.6	17.1	<0.001

STEMI, ST-elevation myocardial infarction; NSTEMI, non ST-elevation myocardial infarction; IQR, interquartile range; BMI, body mass index; AHT, arterial hypertension; DM, diabetes mellitus; DLD, dyslipidaemia; MI, myocardial infarction; CVD, cardiovascular disease; ASA, acetylsalicylic acid; CKD, chronic kidney disease.

**Table 5:** Receiver operator curve (ROC) analysis.

	(A) In-Hospital MACE (*n* = 85)			
	Cutoff	Youden’s Index	Sensitivity/Specificity	AUC
ABI	<0.94	0.146	24.7%/89.9%	0.525
	**(B) All-Cause Deaths [longitudinal] (*n* = 51)**			
	**Cutoff**	**Youden’s Index**	**Sensitivity/Specificity**	**AUC**
ABI	<0.94	0.209	31.4%/89.5%	0.571

MACE, major adverse cardiovascular events; AUC, area under curve.

## Data Availability

All data presented in the study are available to the editors upon request.

## Abbreviations

The following abbreviations are used in this manuscript:

AASI	Ambulatory arterial stiffness index
ABI	Ankle-brachial index
ACC	American College of Cardiology
ACS	Acute coronary syndrome
AHA	American Heart Association
AHT	Arterial hypertension
AMI	Acute myocardial infarction
ASA	Acetylsalicylic acid
AUC	Area under curve
BMI	Body mass index
BP	Blood pressure
CAD	Coronary artery disease
CI	Confidence intervals
CKD	Chronic kidney disease.
CV	Cardiovascular
CVD	Cardiovascular disease
DBP	Diastolic blood pressure
DLD	Dyslipidemia
DM	Diabetes mellitus
EF	Ejection fraction
ESC	European Society of Cardiology
GFR	Glomerular filtration rate
GRACE	Global Registry of Acute Coronary Events
HDL	High-density lipoproteins
HR	Hazard ratios
IQR	Interquartile range
LEAD	Lower extremity arterial disease
LDL	Low-density lipoproteins
MACE	Major adverse cardiovascular events
MI	Myocardial infarction
MSAD	Multisite artery disease
NSTEMI	Non ST-elevation myocardial infarction
OR	Odds ratios
SBP	Systolic blood pressure
STEMI	ST-elevation myocardial infarction
PAD	Peripheral artery disease
TIA	Transient ischemic attack
TG	Triglycerides
UA	Unstable angina

## Appendix A

**Table A1:** Baseline Clinical and Demographic Characteristics According to ABI Status.

	ABI ≤ 0.90 (*n* = 46)	ABI > 0.90 (*n* = 395)	*p* Value
Age, years [median (IQR)]	66 (57 to 78)	61 (54 to 70)	0.04
Sex, % female	26.1	20.0	0.33
BMI [median (IQR)]	28.1 (25.6 to 32.0)	28.4 (25.2 to 32.2)	0.34
Smoking (current), %	47.8	52.9	0.51
Smoking (anytime), %	63.0	65.3	0.76
AHT, %	73.9	66.8	0.32
AHT medication use, %	67.4	61.3	0.42
DM, %	37.0	28.9	0.26
DM medication use, %	32.6	21.8	0.098
DLD, %	67.4	64.8	0.73
Statin use, %	37.0	40.3	0.67
Previous MI, %	26.1	20.5	0.38
CVD family history, %	19.6	28.1	0.22
ASA use, %	37.0	27.3	0.17
Chronic heart disease, %	37.0	27.8	0.20
Heart failure, %	15.2	10.9	0.38
CKD, %	26.1	9.6	<0.001

IQR, interquartile range; BMI, body mass index; AHT, arterial hypertension; DM, diabetes mellitus; DLD, dyslipidaemia; MI, myocardial infarction; CVD, cardiovascular disease; ASA, acetylsalicylic acid; CKD, chronic kidney disease.

**Table A2:** Demographic and Clinical Characteristics According to Acute Coronary Syndrome Type Stratified by ABI.

ABI ≤ 0.9			
	STEMI (*n* = 18)	NSTEMI (*n* = 28)	*p* Value
Age, years [median (IQR)]	63 (59 to 67)	69 (56 to 80)	0.21
Sex, % female	11.1	35.7	0.064
BMI [median (IQR)]	28.0 (26.1 to 33.2)	28.2 (25.4 to 31.4)	0.82
Smoking (current), %	61.1	39.3	0.15
Smoking (anytime), %	72.2	57.1	0.30
AHT, %	66.7	78.6	0.37
AHT medication use, %	61.1	71.4	0.47
DM, %	27.8	42.7	0.30
DM medication use, %	22.2	39.3	<0.23
DLD, %	72.4	64.3	0.58
Statin use, %	27.8	42.9	0.30
Previous MI, %	22.2	28.6	0.63
CVD family history, %	16.7	21.4	0.69
ASA use, %	22.2	46.4	0.097
Chronic heart disease, %	33.3	39.3	0.68
Heart failure, %	5.6	21.4	0.14
CKD, %	5.6	39.3	0.011
**ABI > 0.9**			
	**STEMI (*n* = 224)**	**NSTEMI (*n* = 171)**	***p*** **Value**
Age, years [median (IQR)]	60 (53 to 68)	65 (57 to 74)	<0.001
Sex, % female	17.9	22.8	0.22
BMI [median (IQR)]	28.7 (25.5 to 32.8)	28.3 (24.8 to 31.1)	0.13
Smoking (current), %	57.1	47.4	0.054
Smoking (anytime), %	70.5	58.5	0.013
AHT, %	61.6	73.7	0.012
AHT medication use, %	54.0	70.8	<0.001
DM, %	24.6	34.5	0.031
DM medication use, %	15.2	304	<0.001
DLD, %	61.6	69.0	0.13
Statin use, %	33.9	48.5	0.003
Previous MI, %	32.1	22.8	0.001
CVD family history, %	16.7	21.4	0.041
ASA use, %	17.4	40.4	<0.001
Chronic heart disease, %	20.1	38.0	<0.001
Heart failure, %	7.1	15.8	0.006
CKD, %	6.7	13.5	0.024

ABI, ankle-brachial index; STEMI, ST-elevation myocardial infarction; NSTEMI, non ST-elevation myocardial infarction; IQR, interquartile range; BMI, body mass index; AHT, arterial hypertension; DM, diabetes mellitus; DLD, dyslipidaemia; MI, myocardial infarction; CVD, cardiovascular disease; ASA, acetylsalicylic acid; CKD, chronic kidney disease.

**Table A3:** Hemodynamic, Laboratory and Angiographic Profiles by ACS Type and ABI Status.

ABI ≤ 0.9			
	STEMI (*n* = 18)	NSTEMI (*n* = 28)	*p* Value
SBP at presentation, mmHg [median (IQR)]	133 (124 to 144)	140 (132 to 150)	0.32
DBP at presentation, mmHg [median (IQR)]	87 (75 to 90)	80 (70 to 89)	0.23
HR at presentation, bpm [median (IQR)]	78 (75 to 98)	85 (76 to 97)	0.62
Ejection fraction, % [median (IQR)]	45 (40 to 50)	45 (35 to 55)	0.78
Troponin at presentation, ng/mL [median (IQR)]	905 (204 to 23,220)	850 (74 to 2834)	0.50
Total cholesterol, mg/dL [median (IQR)]	192 (162 to 241)	178 (148 to 223)	0.22
LDL, mg/dL [median (IQR)]	131 (112 to 155)	107 (83 to 151)	0.17
HDL, mg/dL [median (IQR)]	32 (29 to 41)	36 (32 to 44)	0.13
TG, mg/dL [median (IQR)]	157 (105 to 172)	133 (76 to 170)	0.24
Cr at presentation, mg/dL [median (IQR)]	0.9 (0.9 to 1.1)	1.0 (0.8 to 1.5)	0.43
Cr peak, mg/dL [median (IQR)]	1.1 (1.0 to 1.3)	1.2 (0.9 to 1.8)	0.35
Cr on discharge, mg/dL [median (IQR)]	1.0 (0.8 to 1.2)	1.0 (0.8 to 1.4)	0.62
CA LMA findings, %	5.6	25.0	0.089
CA RCA findings, %	61.1	50.0	0.46
CA LADA findings, %	72.2	67.9	0.75
CA LCxA findings, %	38.9	42.9	0.79
CA PDA findings, %	11.1	10.7	0.97
CA DA findings, %	11.1	21.4	0.37
CA OMA findings, %	11.1	14.3	0.76
**ABI > 0.9**			
	**STEMI (*n* = 224)**	**NSTEMI (*n* = 171)**	***p*** **Value**
SBP at presentation, mmHg [median (IQR)]	135 (124 to 162)	140 (130 to 156)	0.045
DBP at presentation, mmHg [median (IQR)]	80 (70 to 90)	86 (76 to 95)	0.017
HR at presentation, bpm [median (IQR)]	78 (70 to 89)	78 (68 to 89)	0.97
Ejection fraction, % [median (IQR)]	45 (37 to 50)	50 (40 to 55)	<0.001
Troponin at presentation, ng/mL [median (IQR)]	1856 (178 to 17,854)	600 (155 to 3310)	0.003
Total cholesterol, mg/dL [median (IQR)]	194 (166 to 234)	181 (148 to 225)	0.003
LDL, mg/dL [median (IQR)]	130 (103 to 160)	115 (77 to 146)	<0.001
HDL, mg/dL [median (IQR)]	39 (33 to 46)	37 (32 to 45)	0.31
TG, mg/dL [median (IQR)]	127 (84 to 172)	138 (109 to 186)	0.014
Cr at presentation, mg/dL [median (IQR)]	0.9 (0.8 to 1.1)	1.0 (0.8 to 1.2)	0.073
Cr peak, mg/dL [median (IQR)]	1.0 (0.9 to 1.2)	1.1 (0.9 to 1.4)	0.018
Cr on discharge, mg/dL [median (IQR)]	0.9 (0.8 to 1.1)	1.0 (0.9 to 1.2)	0.021
CA LMA findings, %	8.0	14.0	0.055
CA RCA findings, %	54.9	55.0	0.99
CA LADA findings, %	67.4	70.2	0.56
CA LCxA findings, %	39.3	47.4	0.11
CA PDA findings, %	8.9	6.4	0.36
CA DA findings, %	15.2	17.5	0.80
CA OMA findings, %	15.2	17.0	0.63

ABI, ankle-brachial index; STEMI, ST-elevation myocardial infarction; NSTEMI, non ST-elevation myocardial infarction; SBP, systolic blood pressure; IQR, interquartile range; DBP, diastolic blood pressure; HR, heart rate; LDL, low density lipoproteins; HDL, high density lipoproteins; TG, triglycerides; Cr, creatinine; CA, coronary angiography; LMA, left main artery; RCA, right coronary artery; LADA, left anterior descending artery; LCxA, left circumflex artery; PDA, posterior descending artery; DA, diagonal artery; OMA, obtuse marginal artery.

**Table A4:** Predictive Value of Arterial Stiffness and ABI for In-Hospital Events.

	(A) MACE (*n* = 85)	
	OR (95% CI)	*p* Value
EF, per 1% increase	0.93 (0.91–0.96)	<0.001
ABI < 0.90	2.93 (1.48–5.81)	0.002
	(B) MI (*n* = 24)	
	OR (95% CI)	*p* value
Age, per 1-year increase	1.06 (1.02–1.10)	0.005
	(C) Cerebrovascular Events (*n* = 11)	
	OR (95% CI)	*p* value
Troponin peak, per 10 ng/L increase	1.11 (1.04–1.18)	0.002
	(D) Arrythmia (*n* = 48)	
	OR (95% CI)	*p* value
EF, per 1% increase	0.92 (0.89–0.96)	<0.001
	(E) Worsening of Renal Function (*n* = 13)	
	OR (95% CI)	*p* value
EF, per 1% increase	0.91 (0.85–0.97)	0.003
	(F) Creatinine Peak	
	beta (SE)	*p* value
EF, per 1% increase	−0.007 (0.002)	<0.001
Creatinine at presentation, per 0.1 mg/dL increase	0.135 (0.033)	<0.001
	(G) Atrial Fibrillation (*n* = 69)	
	OR (95% CI)	*p* value
EF, per 1% increase	0.94 (0.91–0.97)	<0.001
AASI, per 0.1 increase	1.34 (1.12–1.60)	0.002

MACE, major adverse cardiovascular events; OR, odds ratio; CI, confidence intervals; EF, ejection fraction; ABI, ankle-brachial index; MI, myocardial infarction; acute coronary syndrome; SE, standard error.

**Table A5:** Predictive Value of Arterial Stiffness and ABI on longterm outcomes.

	(A) Composite Outcome (*n* = 139)	
	HR (95% CI)	*p* Value
EF, per 1% increase	0.97 (0.95–0.99)	<0.001
AASI, per 0.1 increase	1.21 (1.08–1.35)	0.001
	(B) All-Cause Deaths (*n* = 51)	
	HR (95% CI)	*p* value
EF, per 1% increase	0.91 (0.88–0.94)	<0.001
ABI < 0.90	2.88 (1.53–5.42)	<0.001
	(C) Acute Coronary Syndrome (*n* = 100)	
	HR (95% CI)	*p* value
Troponin peak, per 10 ng/L increase	1.02 (1.01–1.04)	0.002
AASI, per 0.1 increase	1.25 (1.09–1.43)	0.001
	(D) Cerebrovascular Events (*n* = 51)	
	HR (95% CI)	*p* value
Age, per 1-year increase	1.04 (1.01–1.06)	0.005
Troponin peak, per 10 ng/L increase	1.04 (1.02–1.06)	<0.001
	(E) Arrythmia (*n* = 60)	
	HR (95% CI)	*p* value
EF, per 1% increase	0.91 (0.88–0.94)	<0.001
Troponin peak, per 10 ng/L increase	1.04 (1.01–1.06)	0.005
	(F) Worsening of Renal Function (*n* = 8)	
	HR (95% CI)	*p* value
Age, per 1-year increase	1.11 (1.03–1.19)	0.004
	(G) Creatinine Change (exit to 36 m)	
	beta (SE)	*p* value
ABI < 0.90	0.12 (0.04)	0.004

HR, hazard ratio; CI, confidence intervals; EF, ejection fraction; AASI, ambulatory arterial stiffness index; ABI, ankle-brachial index; Acute Coronary Syndrome (recurrent MI and urgent coronary revascularization).
